# Supplementary material for: Direct inference and control of genetic population structure from RNA sequencing data
Source: Commun Biol. 2023 Aug 2;6:804. doi: 10.1038/s42003-023-05171-9 (PMC10397182; doi:10.1038/s42003-023-05171-9)
Supplement: Supplementary file 3 — Description of Additional Supplementary Files [file 42003_2023_5171_MOESM3_ESM.pdf]

Description of Additional Supplementary Files

File Name: Supplementary Data 1
Description: Source data for Figures 2, 4a, and 5
