# Supplementary material for: Direct inference and control of genetic population structure from RNA sequencing data
Source: Commun Biol. 2023 Aug 2;6:804. doi: 10.1038/s42003-023-05171-9 (PMC10397182; doi:10.1038/s42003-023-05171-9)
Supplement: Supplementary file 5 — Reporting Summary [file 42003_2023_5171_MOESM5_ESM.pdf]

nature portfolio

Corresponding author(s): Prof Michael Inouye, Dr Muhamad Fachrul

Last updated by author(s): Jul 20, 2023

Reporting Summary

Nature Portfolio wishes to improve the reproducibility of the work that we publish. This form provides structure for consistency and transparency in reporting. For further information on Nature Portfolio policies, see our Editorial Policies and the Editorial Policy Checklist.

Statistics

For all statistical analyses, confirm that the following items are present in the figure legend, table legend, main text, or Methods section.

n/a	Confirmed	
☐	☒	The exact sample size (*n*) for each experimental group/condition, given as a discrete number and unit of measurement
☐	☒	A statement on whether measurements were taken from distinct samples or whether the same sample was measured repeatedly
☐	☒	The statistical test(s) used AND whether they are one- or two-sided<br>*Only common tests should be described solely by name; describe more complex techniques in the Methods section.*
☐	☒	A description of all covariates tested
☐	☒	A description of any assumptions or corrections, such as tests of normality and adjustment for multiple comparisons
☐	☒	A full description of the statistical parameters including central tendency (e.g. means) or other basic estimates (e.g. regression coefficient) AND variation (e.g. standard deviation) or associated estimates of uncertainty (e.g. confidence intervals)
☐	☒	For null hypothesis testing, the test statistic (e.g. *F*, *t*, *r*) with confidence intervals, effect sizes, degrees of freedom and *P* value noted<br>*Give P values as exact values whenever suitable.*
☒	☐	For Bayesian analysis, information on the choice of priors and Markov chain Monte Carlo settings
☒	☐	For hierarchical and complex designs, identification of the appropriate level for tests and full reporting of outcomes
☐	☒	Estimates of effect sizes (e.g. Cohen's *d*, Pearson's *r*), indicating how they were calculated

Our web collection on statistics for biologists contains articles on many of the points above.

Software and code

Policy information about availability of computer code

Data collection	No notable software was used during data collection.
Data analysis	Analyses were performed using a combination of bash, R, and Python environment. We used a custom pipeline detailed here: https://github.com/fachrulm/RGStraP.

For manuscripts utilizing custom algorithms or software that are central to the research but not yet described in published literature, software must be made available to editors and reviewers. We strongly encourage code deposition in a community repository (e.g. GitHub). See the Nature Portfolio guidelines for submitting code & software for further information.

Data

Policy information about availability of data

All manuscripts must include a data availability statement. This statement should provide the following information, where applicable:

- Accession codes, unique identifiers, or web links for publicly available datasets
- A description of any restrictions on data availability
- For clinical datasets or third party data, please ensure that the statement adheres to our policy

Sequence data associated with this study were deposited to the European Genome-phenome Archive (EGA) under accession number EGAD00001011131. All other data supporting the findings are either presented as Supplementary Data or may be obtained from the authors (MF & MI) on reasonable request.

Human research participants

Policy information about studies involving human research participants and Sex and Gender in Research.

Reporting on sex and gender	Sex (biological attribute) of the participants is part of the included metadata. The findings of this study are relevant on a population-level instead of individual-level as it looks at the differences in population structure between subpopulations of a diverse region, regardless of sex. That being said, genes from sex chromosomes were filtered out prior to our analysis.
Population characteristics	The participants' age ranged from 3 to 80 years old, with the median age being 27 years old. Based on self-reported ethnicity, participants belong to up to 6 broad Nepalese ethnic / caste groups. Participants were recruited for a typhoid surveillance study and made up of three different disease groups: febrile, culture (+); febrile, culture (-); and healthy controls.
Recruitment	Participants were recruited in Latlipur, Nepal as part of the Strategic Typhoid Alliance across Africa and Asia (STRATAA) study, which included passive surveillance for enteric fever and a population-based serosurvey.
Ethics oversight	Nepal Health Research Council (NHRC, ref 306/2015) and OxTREC (Oxford Tropical Research Ethics Committee, ref 39-15)

Note that full information on the approval of the study protocol must also be provided in the manuscript.

Field-specific reporting

Please select the one below that is the best fit for your research. If you are not sure, read the appropriate sections before making your selection.

☒ Life sciences ☐ Behavioural & social sciences ☐ Ecological, evolutionary & environmental sciences

For a reference copy of the document with all sections, see nature.com/documents/nr-reporting-summary-flat.pdf

Life sciences study design

All studies must disclose on these points even when the disclosure is negative.

Sample size	Prior sample-size calculation was not utilized to determine the included samples in this study. We retained all samples that passed quality control filter since our goal is to capture genetic structure from RNA-seq data, and despite there not being a minimum number of sample size for our specific type of analysis, the power of the analysis will only be strengthened by the inclusion of more samples; there is no reason to exclude high-quality samples from this analysis.
Data exclusions	We excluded genes from sex chromosomes per standard procedure when calling genetic variants for population structure purposes.
Replication	The samples used for this study were part of a broader clinical study which took blood sample from each participant at a specific time-point. In variant calling studies each individual is represented by one sample, and the presence of replicates is not desired (replicates, if present, would be merged in such situations).
Randomization	No randomization was done in this study. Blood samples were taken from each participant, each belonging to either one of three disease groups. We take into account participant age and disease group when performing analysis between samples of different sex.
Blinding	Blinding is not relevant to this study as the participant disease group is regressed out during analysis.

Reporting for specific materials, systems and methods

We require information from authors about some types of materials, experimental systems and methods used in many studies. Here, indicate whether each material, system or method listed is relevant to your study. If you are not sure if a list item applies to your research, read the appropriate section before selecting a response.

Materials & experimental systems

n/a	Involved in the study	
☒	☐	Antibodies
☒	☐	Eukaryotic cell lines
☒	☐	Palaeontology and archaeology
☒	☐	Animals and other organisms
☐	☒	Clinical data
☒	☐	Dual use research of concern

Methods

n/a	Involved in the study	
☒	☐	ChIP-seq
☒	☐	Flow cytometry
☒	☐	MRI-based neuroimaging

Clinical data

Policy information about clinical studies

All manuscripts should comply with the ICMJE guidelines for publication of clinical research and a completed CONSORT checklist must be included with all submissions.

Clinical trial registration	ISRCTN 12131979
Study protocol	The STRATAA study protocol can be found here: https://bmjopen.bmj.com/content/7/6/e016283
Data collection	Data collection was done in three different study sites: Dhaka, Bangladesh; Blantyre, Malawi; and Patan, Nepal. The studies were performed in parallel at each of the three chosen field sites, starting in May 2016 with activities continuing until October 2018
Outcomes	Using census-defined denominator populations of ≥100000 individuals at sites in Malawi, Bangladesh and Nepal, the primary outcome is to characterize the burden of enteric fever in these populations over a 24-month period. During passive surveillance, clinical and household data, and laboratory samples were collected from febrile individuals. In parallel, healthcare utilisation and water, sanitation and hygiene surveys were performed to characterise healthcare-seeking behaviour and assess potential routes of transmission. The rates of both undiagnosed and subclinical exposure to typhoidal Salmonellae (seroincidence), identification of chronic carriage and population seroprevalence of typhoid infection will be assessed through age-stratified serosurveys performed at each site. Secondary attack rates will be estimated among household contacts of acute enteric fever cases and possible chronic carriers.
